# Autoinflammatory gene mutations associated with eosinophilia and asthma

**DOI:** 10.1186/s13223-023-00837-9

**Published:** 2023-08-29

**Authors:** Bashayr M. Alotaibi, Raquel Lopez Rodriguez, Carmen Venegas Garrido, Lucia Gonzalez Bravo, Nader Khalidi, Parameswaran Nair

**Affiliations:** 1 Divisions of Respirology, Department of Medicine, McMaster University & St Joseph’s Healthcare, 50 Charlton Ave East, Hamilton, ON L8N 4A6 Canada; 2 Division of Pulmonary Medicine, Department of Medicine, King Saud University Medical City, King Saud University, Riyadh, Saudi Arabia; 3 Divisions of Rheumatology, Department of Medicine, McMaster University & St Joseph’s Healthcare, Hamilton, ON Canada

**Keywords:** Familial Mediterranean fever, Majeed syndrome, Cryopyrin-associated, Autoinflammatory syndrome, Blau syndrome, Genetic testing, Respiratory diseases, Asthma

## Abstract

**Background:**

Respiratory conditions, such as asthma, are infrequently associated with auto-inflammatory diseases. We describe five patients with uncontrolled respiratory symptoms that were seen at St. Joesph’s Healthcare in Hamilton for severe asthma management diagnosed with rare autoinflammatory conditions using genetic molecular analysis.

**Case presentation:**

Five patients are included in this case series. Gene mutations associated with familial Mediterranean fever, Yao syndrome, Cryopyrin-associated periodic syndrome, and Majeed syndrome were considered to explain partly the patient’s clinical manifestation after comprehensive clinical, biochemical, hematological investigations ruled out other disorders such as parasitosis, Allergic Bronchopulmonary Fungosis, Eosinophilic Granulomatosis with Poly Angitis, IgG4 disease, and Hypereosinophilia syndrome.

**Conclusions:**

Complex patients initially presenting with respiratory conditions in addition to unexplained autoinflammatory conditions are a diagnostic challenge. Genetic molecular testing provides healthcare practitioners with useful information that may diagnose underlying auto-inflammatory diseases in undifferentiated patients. Role of inflammasome-activation in asthma and eosinophilia needs further investigation.

## Background

Severe asthma is characterized by ongoing asthma symptoms and poor response to standard treatment driven by a biological phenomenon that is truly refractory to usual treatment and requires higher intensity therapies, whereas difficult-to-treat asthma is a broader term that can include patients with a range of asthma subtypes that are hard to manage [1]. These difficult-to-treat asthma can be due to concomitant disorders which could over-estimate the severity of asthma, including vocal cord dysfunction, tracheobronchomalacia and intercurrent infections. Eosinophilia (in peripheral blood or in sputum) is often considered as a surrogate for the severity of asthma [2]. This case series describes a rare group of disorders, collectively termed autoinflammatory syndrome, as another co-morbidity that could be associated with asthma and eosinophilia.

## Case presentations

### Case 1

A 56 years-old female with multiple environmental allergies was referred to our clinic for evaluation for asthma as a possible explanation for her ongoing respiratory symptoms despite high doses of inhaled corticosteroids and long-acting bronchodilators with rapid improvement on salbutamol and oral prednisone. She had been diagnosed with psoriatic arthritis based on cutaneous lesions, enthesitis, and positive HLA B27, for which she had received a short course of treatment with azathioprine, methotrexate, and a high dose of prednisone. She also had symptoms of morning fever (3 times/week), abdominal, joint, and whole-body pain, morning stiffness, episodic hematuria, intermittent scaly rashes, intense photosensitivity, oral ulcers, and sicca symptoms. A skin biopsy revealed lymphocytic-eosinophilic infiltrates suggestive of contact dermatitis. Carbon monoxide diffusion coefficient, inspiratory and expiratory mouth pressures and diaphragm assessment by ultrasonography, performed to assess a non-obstructive spirometry, were normal. Chest computed tomography scan showed mild (12–14 mm) mediastinal and hilar lymphadenopathy. Endobronchial ultrasound guided needle aspiration showed only reactive adenopathy. Genetic analysis revealed the presence of a heterozygote variant (C.44 2G > C, p. Glu14 AGIN) in MEFV gene, consistent with familial Mediterranean fever (FMF).

### Case 2

A 47-year-old male was referred for assessment of possible Eosinophilic Granulomatosis with Polyangitis on the background of peripheral eosinophilia (peak 0.9 × 10^9^/L), axillary lymph node enlargement, skin rashes, mild naso-bronchial allergy (peak serum IgE > 5000 KU/L) and arthralgia. Spirometry was normal. He had borderline normalairway hyperresponsiveness (Provocative Concentration of methacholine-PC20 14 mg/ml, tidal breathing method) [3]. Additional focused history included recurrent waxing and waning painful lymphadenopathies from childhood affecting the axillary, inguinal, cervical, and clavicular regions. Excision lymph node biopsy had shown only reactive changes; IgG4 staining was negative. He also reported a self-limiting erythematous and itching skin rash that lasted for one week, along with occasional conjunctivitis.The only abnormality on clinical examination, in addition to small mobile firm axillary lymph nodes, was mild hepatosplenomegaly. Patient did not meet the criteria for Eosinophilic Granulomatosis with Polyangiitis (EGPA). Genetic analysis revealed the presence of two heterozygote variants detected in the NOD2 gene, (c.2104 C > T, p. Arg702Trp) and (c.2798 + 58 C > T), fulfilling 3/4 criteria for Yao syndrome. He was started on Colchicine with disease stabilization and weaned himself off his inhaled corticosteroid and long-acting beta-agonist.

### Case 3

A 43-year-old female was seen for evaluation of marked eosinophilia (peak 5.8 × 10^9^/L) in the context of intermittent recurrent fever, fatigues, urticaria rash, adenopathy, chest pain, dyspnea, and pleuritis, since January 2022 (18 months) She had previous documentation of positive serology for strongyloidiasis and had received two courses of ivermectin. She had mild airway hyperresponsiveness (PC20methacholine 10.8 mg. ml) with normal spirometry (suggesting that current symptoms due to asthma i.e. variable airflow obstruction, were likely to be absent or mild), and chronic spontaneous urticaria that had responded to rupatadine. There was no evidence of increased mast cell activity (normal serum and sputum tryptase). Her bone marrow biopsy, flow cytometry, and needle core biopsy of the left axillary lymph node were normal, ruling out hematologic malignancy and hypereosinophilic syndrome. Genetic analysis revealed the presence of two heterozygote variants (c.598G > A, p. Val200Me) in NLRP3 gene, consistent with a diagnosis of Cryopyrin-associated Periodic Syndrome (CAPS), and was started on Colchicine that has normalized her blood eosinophil count. Her asthma is also very well controlled on low dose of inhaled corticosteroid and long-beta agonist.

### Case 4

A 35-year-old female with a history of atopy (cat allergy, peak total serum IgE 567 KU/L, peak blood eosinophil 0.7 × 10^9^/L) and asthma (mild airflow obstruction and moderate increase in airway hyperresponsiveness PC20 methacholine 0.44 mg/ml) since childhood controlled with moderate doses of inhaled corticosteroid and long acting beta2 agonist developed during two pregnancies, scleritis, recurrent arthralgias, skin rash, oral ulcers, and lower back pain, without facial swelling, neutrophilic dermatosis, or photosensitivity. Both babies were born without complications. Her cardiac and pulmonary examination were within normal limits. Genetic testing was positive for periodic fever syndrome, showing a heterozygote variant of *LPIN2* gene *c.1510 C > T (p.Leu504Phe).* Her mother also had the same mutation. Her asthma is well controlled, but she continues to have recurrent arthralgias and fevers, and has deferred taking Anakinra or Colchicine that were recommended. She does not meet the clinical criteria for Majeed’s syndrome, but the mutations are likely to account partly for her ongoing symptoms.

### Case 5

A 43-year-old male was referred for evaluation of severe asthma, chronic rhinosinusitis with polyps, unexplained fatigue, malaise and unexplained eosinophilia (peak blood 1.2 × 10^9^/L, peak sputum 48.5%). Comprehensive neuromuscular evaluation, including myositis autoimmune panel, Electromyography, and muscle Magnetic Resonance Imaging studies did not show abnormalities. His asthma and eosinophilia were well controlled with benralizumab. Genetic testing revealed a mutation of the MEFV gene c442G > C (p.Glu148Gl) that was considered a variant of uncertain significance. (Tables 1 and 2)

**Table 1:** Results of investigations- respiratory Measurements

	Case 1	Case 2	Case 3	Case 4	Case 5
FEV1(L) FEV1 (%)	1.52 85	5.30 128	2.40 76	2.60 78	3.53 82
FVC (L) FVC (%)	1.82 54	7.21 138	3.02 78	3.60 92	4.42 82
FEV1/FVC %	84	73	79	65	77
Reversibility (%)	9–12	3	3	22	12
PC20 methacholine (mg/ml)	Not done	14	10.8	0.44	1
Skin prick test (# of + ve wheal and flare reactions)	8 (HDM x 2, tree, grass, ragweed, Alternaria, cat, dog)	1 (grass)	None	1 (cat)	none
Blood eosinophil count (10^9^/L) (< 0.4 × 10^9^/L))	0.67	0.9	5.8	0.7	1.2
FeNO (ppb) (< 25ppb)	11	60	32	44	84
Sputum eosinophil (%) <2.2%)	1.3	1.2	1.3	17.8	48.5
IgE (KIU/L) (< 87KIU/L)	1240	> 50,000	292	567	340
Tryptase (ug/L) (< 11.4 ug/L)	4.7	2.6	10.2	3.8	2.2

**Table 2:** Results of investigations- non-respiratory Measurements

	Case 1	Case 2	Case 3	Case 4	Case 5
Quantitative Igs	Normal	Normal	Normal	Normal	Normal
ANA	1:160 (fine speckled)	1:40	1:40	1:40	1:40
RF (IU/mL) (< 20IU/ml)	16	< 10	< 10	< 10	< 10
Anti-CCP (units) (< 20 units)	20	< 18	< 18	< 18	< 18
DsDNA (IU/mL) (< 8IU/ml)	1	1	1	1	1
ANCA	< 0.2	< 0.2	< 0.2	< 0.2	< 0.2
Complements (C3, C4)	Normal	0.82, 0.13	Normal	Normal	Normal
CK (U/L) (< 275U/L)	46	20	20	10	138
ESR (mm/hr) (< 20 mm/hr)	71	2	120	32	2
CRP (mg/L) (< 10 mg/L)	20.8	0.6	198	56	54.5
Ferritin (ug/L) (< 272 ug/L)	64	105	2492	120	110
Infectious serology	Negative	Negative	Negative	Negative	Negative
Stool examination	Normal	Normal	Normal	Normal	Normal
Bone marrow, cytogenetics	Normal	Normal	Normal	Normal	Normal
Genetic results	(C.44 2G > C, p. Glu14 AGIN) in MEFV gene	(c.2014 C > T, p. Arg702Trp) and (c.2798 + 58 C > T) in NOD2 gene	(c.598G > A, p. Val200Me) in NLRP3 gene	c.1510 C > T (p.Leu504Phe) in LPIN2 Gene	MEFV: c442G > C (p.Glu148Gl)
Asthma therapy	Budesonie + Fomoterol DPI	Budesonide + Formoterol DPI PRN	Budesonide + Formoterol DPI PRN	Budesonide + Formoterol DPI	Momentasone + Formoterol MDI benralizumab
Autoinflammatory therapy	Colchicine	Colchicine	Colchicine	Anakinra recommended	Colchicine recommended

IgE, immunoglobulin E; Igs, immunoglobulin; ANA, antinuclear antibodies; RF, rheumatoid factor; anti-CCP, anti-cyclic citrullinated peptide; dsDNA, double-stranded DNA; ANCA, anti-neutrophil cytoplasmic antibodies; CK, creatine kinase; ESR, erythrocyte sedimentation rate; CRP, C-reactive protein. Normal values are provided within parentheses in column 1

## Discussion and conclusions

We present five patients suspected of severe asthma and peripheral eosinophilia, all of whom had genetic mutations associated with auto-inflammatory diseases that partly explained their ongoing symptoms. Asthma was not the major contributor to their ongoing symptoms as evidenced by mild-borderline airway hyperresponsiveness, and normal sputum eosinophils in three of the five patients. This case series shows the growing importance of exome sequencing and genomic medicine in tertiary clinics in evaluation of diverse symptoms associated with eosinophilia and Th2 immune responses.

Auto-inflammatory diseases are characterized by fever and systemic inflammation, which can lead to significant morbidity and mortality [4].Their etiology is explained by aberrant innate immune activation, with varying profiles of upregulated cytokines [5]. FMF is among the most common monogenic disorders, characterized by recurrent fevers and chest, abdominal, or joint pain. Diagnosis involves genetic testing showing *MEFV* gene mutation [6]. Yao syndrome is a polygenic auto-inflammatory disease characterized by fever, dermatitis, polyarthritis, and sicca-like symptoms [7]. Most patients have the *NOD2* variant IVS8^+ 158^, and some carry the R702W variant. CAPS is a spectrum of auto-inflammatory diseases linked to defects in the protein cryopyrin and NLRP3 and 1 inflammasomes [8]. Symptoms such as fever, urticarial rash, and conjunctivitis may be triggered by cold exposure. Majeed syndrome is a rare multi-system auto-inflammatory disorder characterized by neutrophilic dermatosis, multifocal osteomyelitis, dyserythropoietic anaemia etc. caused by mutations in *LPIN2* [9], a gene responsible for producing protein lipin-2, suspected to be involved in fat metabolism.

**Fig. 1:**
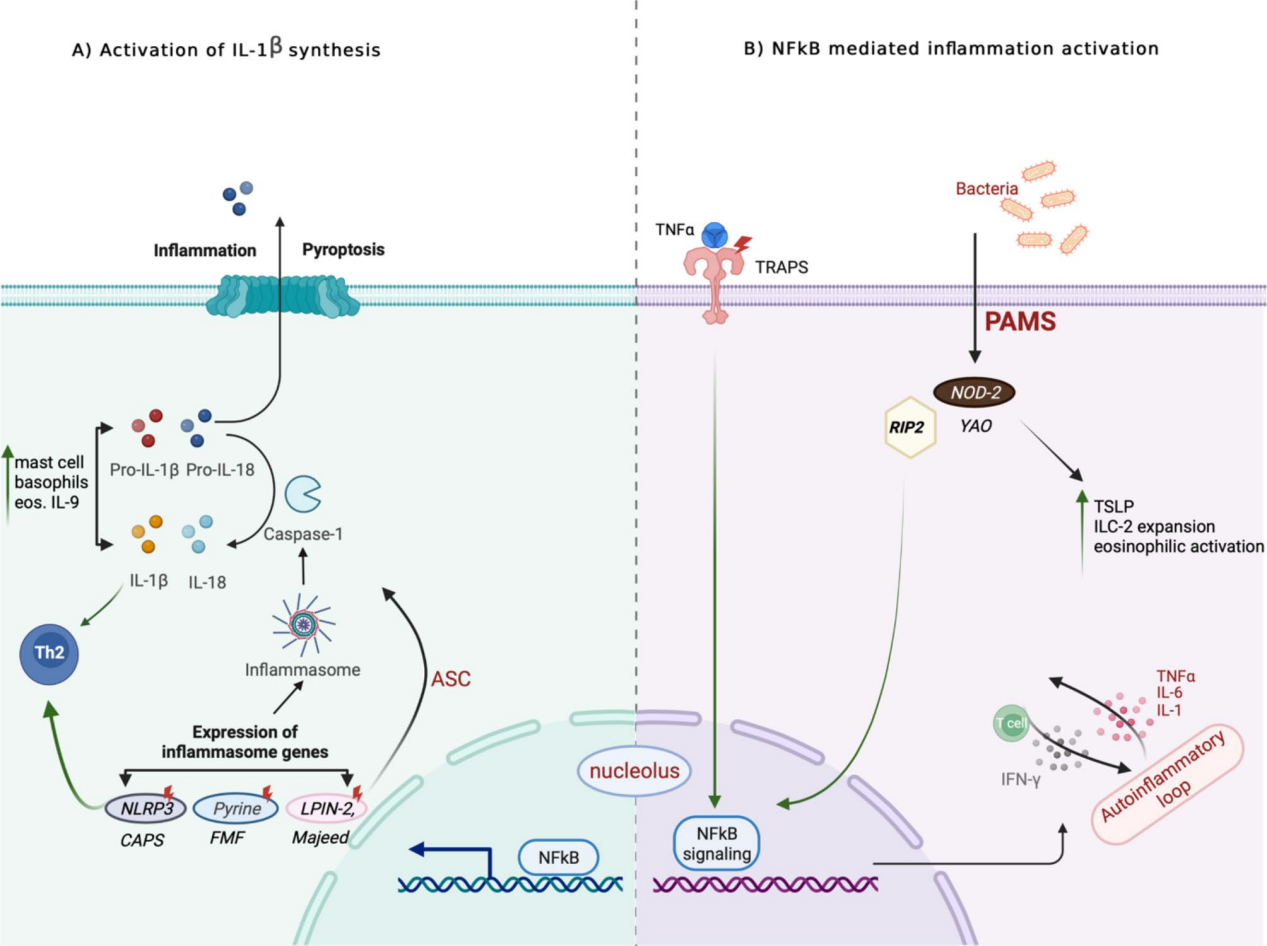
Th2-autoinflammatory pathways interaction: (**A**) Activation of the inflammasome genes including NLRP3, Pyrine, LPIN-2 transform inactive pro-IL-1b and pro-IL-18 into their active forms which lead to enhanced Th2 differentiation. IL-1b induce allergic lung inflammation via different effector cells, while IL-18 has context-dependent effect as it can constrain antiparasitic responses and also induce IL-13 in natural killer (NK) cells and basophils which contribute to the innate part of type 2 immune response. NLRP3 has both inflammasome-dependent and independent pathways which act as brake on type 2 responses. Pyrine has no major role in type 2 immune response, it lessens allergic inflammation by inhibiting NF-kB activation. **(B)** NF-kB signaling is known to play a role in Th2 differentiation and IgE production. TNFa receptors act as major activators to NF-kB pathway, enhances Th2 and Th9 differentiation, and the effect of IL-4 on eosinophils which may worsen asthma. NOD2 enhances TSLP, ILC2, and eosinophilic activation, and constitutive activation of NF-kB leading to upregulation of proinflammatory cytokines. NF-kB, nuclear factor kappa B; IL-1; TRAPS, periodic necrosis factor associated receptor 1; FMF: familiar Mediterranean fever; CAPS: cryopyrin-associated periodic syndrome

There is increasing evidence of an association between auto-inflammatory diseases and type 2 immune responses (Fig. 1). These might be by activation of the inflammosome pathways and involvement of various proteins and cells such as IL-1β, IL-18, NF-κB, ILC2 cells [5–8]. Our patients had heterozygous mutations and those that were considered as variants of uncertain significance. We are not claiming that the patients have the diseases associated with the mutations, but their clinical histories were consistent with what is expected in those diseases, and the eosinophilia might be partly driven by inflammasome activation. Three of the five patients had significant improvement in their eosinophilia and symptoms following treatment with colchicine. We suggest that autoinflammatory conditions may be suspected in those with persistently raised unexplained C-reactive protein (4 of the 5 patients) and characteristic symptoms. The importance of recognizing genetic causes of disease was recently highlighted [10]. The authors recommended genetic testing (targeted panels or whole exome sequencing, as appropriate) in patients with any of the following clinical features: multiple hospital visits and investigations without a unifying pathology, atypical response to conventional treatment, multiple diagnoses that are seemingly unrelated on personal or family history, an illness in a patient of a different demographic than is typical, or a patient lacking the expected risk factors for their presentation. In our clinical practice, we consider this in patients with idiopathic hypereosinophilia [11] or patients with unexplained airway infections often associated with a Th2 immune response.

In conclusion, we describe five rare auto-inflammatory gene mutations in patients referred for assessment for asthma and eosinophilia. Inflammasome activation is recognized as being activated in severe airway diseases associated with neutrophilia [12]. Targeting these pathways in specific eosinophilic conditions and the intersection with Th2 immune pathways warrant more investigation.

## Data Availability

All data supporting the findings of this study are available within the paper and its Supplementary Information.

## Abbreviations

CAPS: Cryopyrin-associated Periodic Syndrome
EGPA: Eosinophilic Granulomatosis with Polyangiitis
FMF: Familial Mediterranean Fever
HLA: Human Leukocyte Antigen
PC20: Provocative Concentration required to cause 20% drop in FEV1
